# Mathematical-Physics Analyses of the Nozzle Shaping at the Aperture Gas Outlet into Free Space under ESEM Pressure Conditions

**DOI:** 10.3390/s24113436

**Published:** 2024-05-26

**Authors:** Pavla Šabacká, Jiří Maxa, Jana Švecová, Jaroslav Talár, Tomáš Binar, Robert Bayer, Petr Bača, Petra Dostalová, Jiří Švarc

**Affiliations:** 1 Faculty of Electrical Engineering and Communication, Brno University of Technology, Technická 10, 616 00 Brno, Czech Republicbinar@vut.cz (T.B.); robert.bayer@vut.cz (R.B.); 186111@vut.cz (P.D.); 2 Institute of Scientific Instruments of the CAS, Královopolská 147, 612 64 Brno, Czech Republic; 3 Faculty of Military Leadership, University of Defence, 662 10 Brno, Czech Republic; jana.svecova@unob.cz (J.Š.); jiri.svarc2@unob.cz (J.Š.)

**Keywords:** Ansys Fluent, ESEM, critical flow, nozzle, CFD, electron dispersion, shock wave

## Abstract

The paper presents a methodology that combines experimental measurements and mathematical-physics analyses to investigate the flow behavior in a nozzle-equipped aperture associated with the solution of its impact on electron beam dispersion in an environmental scanning electron microscope (ESEM). The shape of the nozzle significantly influences the character of the supersonic flow beyond the aperture, especially the shape and type of shock waves, which are highly dense compared to the surrounding gas. These significantly affect the electron scattering, which influences the resulting image. This paper analyzes the effect of aperture and nozzle shaping under specific low-pressure conditions and its impact on the electron dispersion of the primary electron beam.

## 1. Introduction

This paper deals with the issue of pumped vacuum chambers of environmental scanning electron microscopy (ESEM), and its content builds on and expands the area of research from the published paper [1]. In general, electron microscopy allows for a more detailed examination of samples than conventional optical microscopy [2,3]. However, electron microscopy requires a vacuum to allow the primary electron beam to pass through, consequently making it impossible to observe wet samples, as they dry out quickly. Wet sample observation in a conventional electron microscope requires demanding preparation [4]. For this reason, the environmental scanning electron microscope (ESEM) was developed. Here, the specimen chamber is separated from the vacuum areas using an aperture system. It makes it possible to observe wet samples without special preparations and nonconductive or semiconducting samples [5]. Furthermore, it is possible to observe native samples [6] in ESEM without damaging them or samples in dynamic conditions of in situ experiments [7,8]. Special ionization or scintillation detectors detect secondary electrons in ESEM [9,10].

In this paper, the issue of pumping the specimen chamber and the differentially pumped chamber was solved. Dr. Danilatos has been dealing with this issue for a long time [11,12,13]. It is possible to mention some of his publications where it was drawn from.

In the article GAS-FLOW FIELD IN THE ENVIRONMENTAL SEM, the author writes that a conically shaped PLA is preferred for several reasons; one such reason is that it allows the preservation of a stagnation pressure environment over the specimen surface for a short distance down to one PLA diameter. From the number of density contours, no effect is observed over the specimen surface. The same observation is made from speed, temperature, and surface pressure determinations [14,15]. His next work has established that a thin-plate PLA assembly produces the minimum electron beam current loss at the high-vacuum–high-pressure boundary. This was expected because the thin PLA walls result in the most abrupt change in pressure, which generates the steepest gas density decrease along the axis. The thicker PLA design creates a slower pressure transition, which leads to a greater loss in beam current above the PLA [16,17,18]. These considerations are developed by Dr. Danilatos in his other works.

These chambers are separated by a small aperture so that it is possible to maintain the pressure gradient during pumping, where the operating pressure in the specimen chamber is in the order of about 2000 Pa (it can be less or more), and the differential pumped chamber, where the pressure is at least an order of magnitude lower [19,20]. There are high demands on this aperture [21]. Its role is to maintain the pressure gradient between the two chambers. Furthermore, to model the gas flow so that, above this aperture in the differential chamber, where the gas flow is directed due to the pressure gradient, the values of state quantities are such that there is as little scattering of electrons as possible [22,23]. This results in the primary electron beam interacting with gas molecules on its path through the differential pumped chamber to the specimen chamber. Any loss of electrons causes reduced sharpness of the final image. A given aperture is a small and short cylindrical hole. Due to the pressure gradient on this aperture, a critical flow with supersonic velocities occurs in the differentially pumped chamber. The aperture is, therefore, equipped with a nozzle to model the supersonic gas flow, which adjusts the flow of the given supersonic gas flow. The influence of nozzle openings on the distribution of state variables in a given flow and their impact on the primary electron beam scattering was analyzed in the previous article [1]. In this paper, the character of the bottom part opening of the nozzle and its effect on electron beam scattering will be analyzed.

In the presented literature, research is conducted concerning the influence of nozzle angle and thickness on electron beam dispersion. The research presented in this paper builds on the results and expands them with a wider range of nozzle shaping options, which significantly affects gas expansion control. This expansion control has a double effect on the electron beam dispersion. The expansion regulation affects the Mach number and, thus, the pressure course in the primary beam path and the oblique shock wave character. The control of the oblique shock wave shapes is directly related to the formation or suppression of the perpendicular shock wave. It can create an unsuitable pressure barrier for the electrons of the primary beam passing through.

## 2. Effect of the Shape of the Bottom Part of the Nozzle

**Figure 1:**
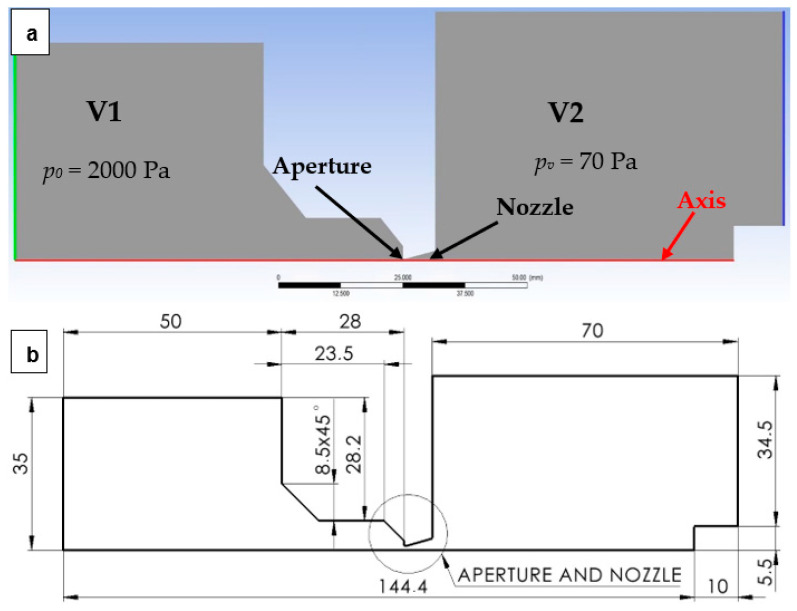
Two-dimensional axisymmetric model of experimental chamber with boundary conditions (**a**); geometry drawing of simulated model (mm) (**b**).

This entire research is conducted on the experimental chamber that simulates the state of flow between the specimen chamber (marked V1 in Figure 1a) and the differentially pumped chamber (marked V2 in Figure 1a) [24]. These chambers are separated by the aperture and nozzle (Figure 1b) in the experimental chamber. This part is designed in the experimental chamber as an exchangeable part.

**Figure 2:**
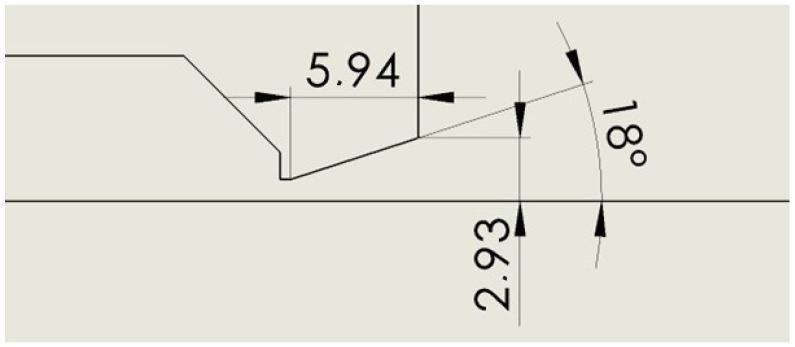
Nozzle angle 18° variant (Nozzle 18).

The opening shapes of the nozzle lower part were compared with the variant that came out the most advantageous for electron scattering in the article [1], therefore, having a nozzle with an opening of 18° (Figure 2).

The issue of the impact of the change in the nozzle angle on the supersonic flow character was analyzed in this paper. This makes it possible to regulate the pressure course on the primary beam path. This is also about the nature of the shock waves. All this leads to a positive effect on the resulting primary electron beam dispersion. In this paper, the shape of the nozzle was first analyzed as a benchmark on a fine-tuned mathematical-physics model with dimensions determined using the theory of one-dimensional isentropic flow for a calculated cross-section with an angle of 12° (Figure 1b). Then, this calculated nozzle condition was compared with the flow of under-expanded nozzles with aperture angles of 8° and 10° and variants with overexpanded nozzles with opening angles of 14°, 16°, and 18°. In the end, the results clearly showed that the shape of the overexpanded open nozzle is more advantageous for the course of the primary electron beam. Here, the flow character with a rapid and very high increase in velocity in the nozzle immediately after the aperture creates a large pressure drop. Although the re-increase in pressure occurs earlier than with under-expanded nozzles, more open nozzles are favorable for the overall environment, which provides conditions for the lowest possible electron beam dispersion. For this reason, this shape was chosen as the default benchmark and compared with other types of nozzles.

**Figure 3:**
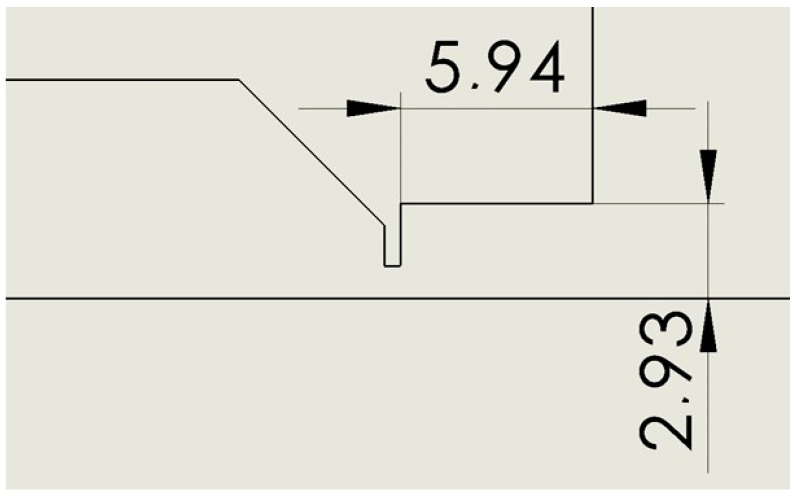
Nozzle cylinder variant (Cylinder).

**Figure 4:**
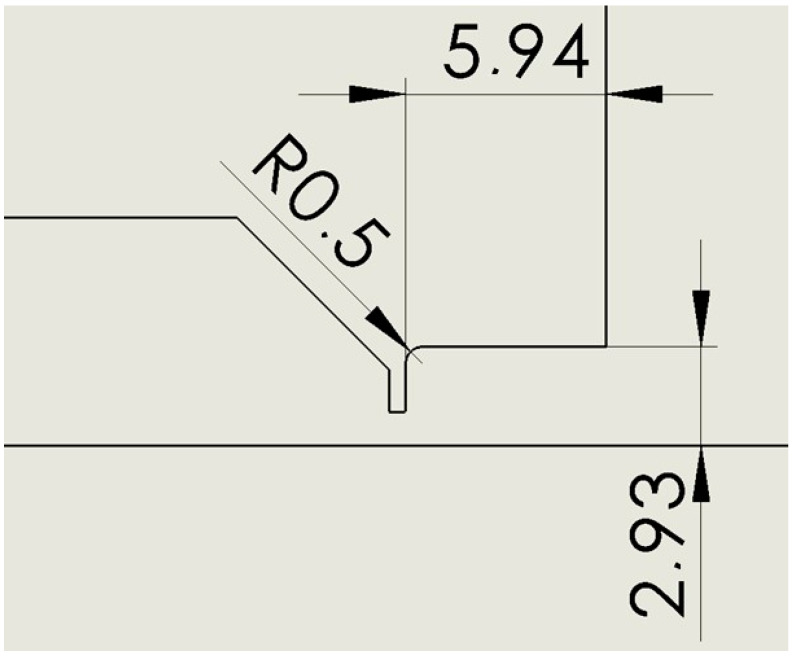
Nozzle cylinder variant with radius of 0.5 mm (R 0.5).

**Figure 5:**
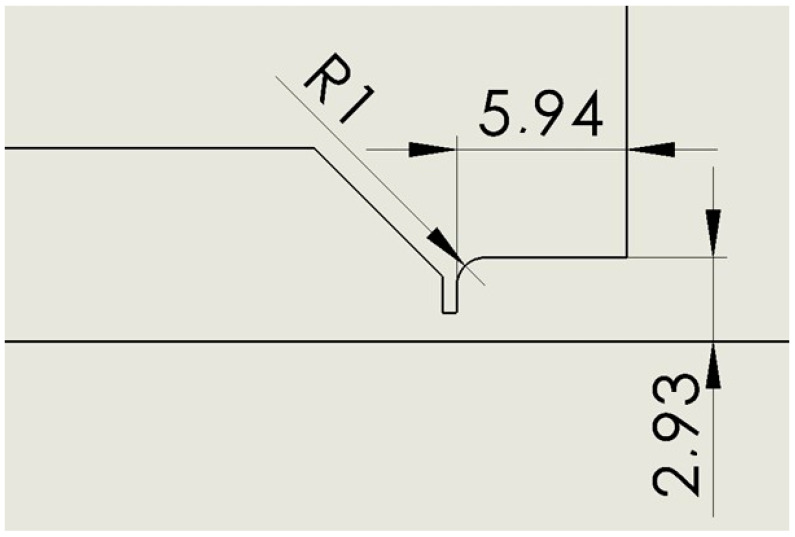
Nozzle cylinder variant with radius of 1 mm (R 1).

**Figure 6:**
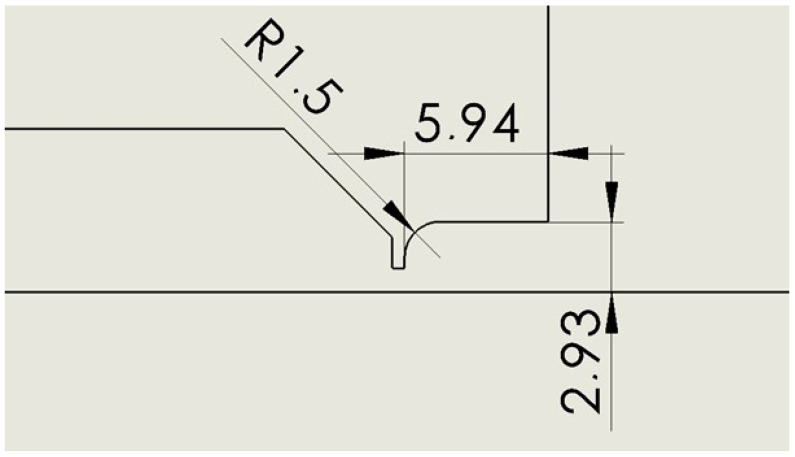
Nozzle cylinder variant with radius of 1.5 mm (R 1.5).

**Figure 7:**
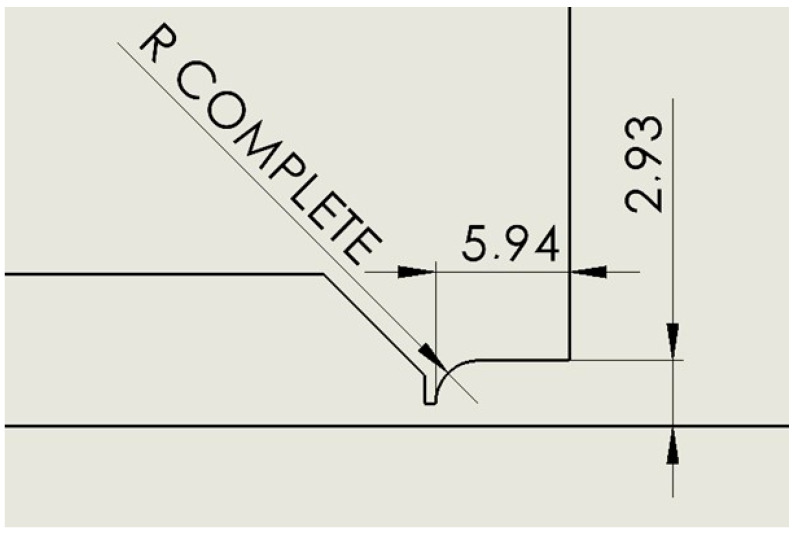
Nozzle cylinder variant with completely rounded radius (R Complete).

**Figure 8:**
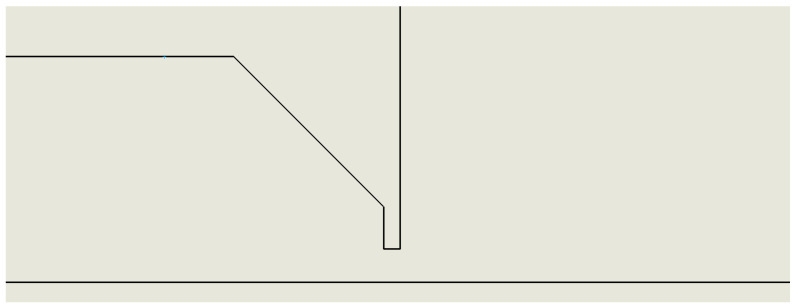
Nozzle-only aperture variant (Open).

All variants have an aperture with a diameter of 2 mm and a length of 0.5 mm. The following variants were compared with each other:

- Nozzle angle 18° (Figure 2).
- Nozzle cylinder (Figure 3).
- Nozzle cylinder with R 0.5 (Figure 4).
- Nozzle cylinder with R1 (Figure 5).
- Nozzle cylinder with R 1.5 (Figure 6).
- Nozzle cylinder with R COMPLETE (Figure 7).
- Nozzle-only aperture (Open) (Figure 8).

### 2.1. Variant: Nozzle Angle 18°

As mentioned above, this variant came out of the previous analyses [1] as the most advantageous for the passage of the primary electron beam when the nozzle was opened at 18°. This variant is compared in this article with variants different from the Laval nozzle and has an open lower part of the nozzle (Figure 2).

### 2.2. Variant: Cylinder

This is a variant where the nozzle is fully open in the shape of a cylinder with a diameter corresponding to the output cross-section of the nozzle with an angle of 18° (Figure 3).

### 2.3. Variant: Cylinder R 0.5

This is a variant where the cylinder variant is rounded with a small radius of 0.5 mm to eliminate possible corner swirling (Figure 4).

### 2.4. Variant: Cylinder R 1

This is a variant that rounds the cylinder variant with a radius of 1 mm to eliminate possible corner swirling (Figure 5).

### 2.5. Variant: Cylinder R 1.5

This is a variant where the cylinder variant is rounded with a radius of 1.5 mm to eliminate possible corner swirling (Figure 6).

### 2.6. Variant: Cylinder Complete

This is a variant where the cylinder variant has a completely rounded radius to eliminate any corner swirling (Figure 7).

### 2.7. Variant: Only Aperture (Open)

The last variant for comparison is a variant where the nozzle is absent and the chambers are separated only by the aperture (Figure 8).

A flow analysis at the pressure gradient was performed from chamber V1, where the boundary conditions of the static pressure *p_o_* = 2000 Pa and the boundary condition of the static pressure *p_v_* = 70 Pa were set in the V2 chamber. It is a pressure gradient with a ratio of 0.035 (Equation (1)) [25]:

(1) $$\frac{P_{v}}{P_{o}}=0.035$$

where *p_0_* is the static pressure in the specimen chamber and *p_v_* is the static pressure in the differentially pumped chamber.

Under the given conditions, according to the theory of one-dimensional isentropic flow, the expected value of the Mach number for the computational nozzle state from Equation (2) [25] is:

(2) $$\frac{p_{v}}{p_{o}}={\left[\frac{2}{2+\left(\varkappa -1\right){Ma}^{2}}\right]}^{\frac{\varkappa }{\varkappa -1}}$$

where *Ma* is the Mach number (*Ma* = 2.83) and *ϰ* is the gas constant (*ϰ* = 1.4).

## 3. Methodology

### CFD Simulation Settings

The nozzle angle of 18° was established as the most advantageous variant for the passage of the primary electron beam using the principles of one-dimensional isentropic flow theory [1]. This configuration ensures controlled gas expansion behind the nozzle [26,27,28,29,30].

Subsequently, the flow behavior within this nozzle was analyzed, employing a tuned mathematical-physics model. This analysis was conducted with the nozzle set at 18° angle for the Laval nozzle [31]. Comparisons were made with alternative opening angles.

Lastly, the findings were assessed in the context of electron scattering analysis, a critical factor influencing microscope functionality. These results serve as fundamental insights for designing differentially pumped chambers.

The application of the theory of one-dimensional isentropic flow anticipates significant pressure and temperature gradients [32,33]. Therefore, the density-based solver setting was selected for analysis as the best solver for this type of analysis. This solver operates on the density-based equations for continuity, momentum, energy, and substance transport simultaneously, with other scalar equations solved sequentially.

The density-based solver solves the governing equations of continuity, momentum, energy, and transport of species simultaneously. The governing equations for other scalars will be separated from each other and from the coupled set. Because the governing equations are nonlinear, several iterations before obtaining a converged solution require a loop of the solution. Each iteration consists of the following steps:

- Update the fluid properties based on the current solution.
- Solve continuity, momentum and energy equations, and species at the same time.
- Where appropriate, solve equations for scalars, such as turbulence and radiation, using previously updated values for the other variables.
- If interphase coupling is to be included, update the source members in the appropriate continuous phase equations by calculating the discrete phase trajectory.
- Check the convergence of the system of equations.
- These steps continue until the convergence criteria are met.

Given the intricate flow dynamics within the nozzle, an implicit linearization approach for solving conjugate equations was deemed optimal. This method linearizes each equation within the set implicitly concerning all dependent variables. The pooled implicit approach, resolving all variables across cells simultaneously, demonstrated stability and suitability for handling the complex supersonic flow conditions and substantial pressure differentials encountered during experimentation with the experimental chamber.

Due to the nature of supersonic flow, it was necessary to use the equation of friction dissipation. Therefore, it was decided to switch the terms of viscous dissipation into the energy equation. Viscous heating is important when the Brinkman number (*Br*) approaches or exceeds unity [34].

(3) $$B_{r}=\frac{\mu U_{e}^{2}}{k\Delta T}$$

where ΔT is the temperature difference in the system.

In the subsequent phase, the Advection Upstream Splitting Method (AUSM) scheme was chosen for the setup. This enhanced approach formulates convective and compressive flows by utilizing the eigenvalues of the Jacobian flow matrices.

The AUSM boasts several advantageous features:

- Accurate representation of shock and contact discontinuities.
- Provision of entropy-conserving solutions.
- Elimination of the “carbuncle” phenomenon, an instability in shock representation often observed in low-dissipative numerical techniques.
- Consistent accuracy and convergence rates across all Mach numbers.

Notably, the method’s efficacy is not contingent upon explicit knowledge of eigenvectors, rendering it especially appealing for systems with unspecified eigenstructures, such as the two-fluid equations governing multiphase flow [34,35].

For inter-cell result transfer, a second-order upwind scheme was implemented. This scheme calculates variables on cell surfaces using a multivariate linear reconstruction approach. By leveraging the Taylor series expansion of a cell-centered solution around the cell’s centroid, higher-order accuracy is achieved at cell faces [36,37,38].

This approach effectively handled the dynamic flow changes induced during pumping, aligning well with experimental measurements. The mathematical-physics analysis necessitated a carefully selected mesh [39].

**Figure 9:**
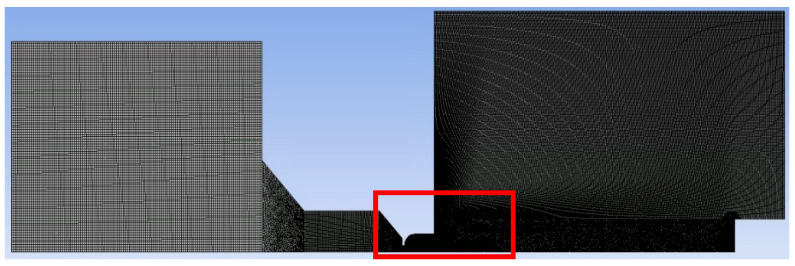
Structured mesh for the mathematical-physics analysis.

A structured mesh combined with a 2D variant of hexagonal elements was utilized, offering advantages such as reduced artifacts from transmitting results over oblique edges and a minimized cell count for purely rectangular regions (Figure 9). In areas where a structured mesh was impractical, triangular elements were employed, notably in narrow sections like the nozzle aperture and regions with anticipated supersonic flow behind the nozzle.

**Figure 10:**
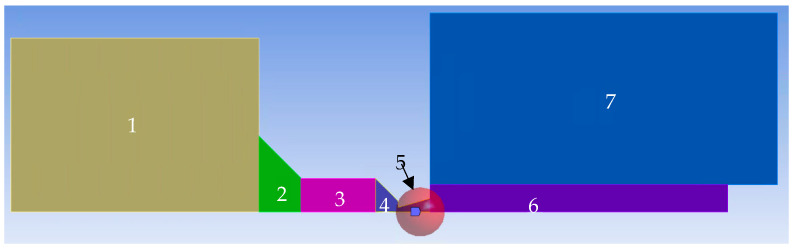
Mesh settings.

The basic mesh settings on which the calculation was based are shown in Figure 10. The computational area has been divided into the following areas to set each with a different mesh fineness and also to separate the areas that can be meshed with a structured mesh from the areas where triangles must be used. Each region has the following settings (Figure 10):

1. Setting up the mesh in a place where there is no complex flow and the pressure is higher than in other areas. It is set as coarsest to 0.4 mm with the Behavior HARD option. Due to the rectangular area, a structured mesh is used.
2. Also, there is no complex flow in this area yet and the pressure is higher than in the other areas. The cell size is set to 0.3 mm with the Behavior SOFT option. Due to the shape-transition area, mesh with triangular elements is used.
3. This area is adjacent to the area where the aperture entrance is already taking place. Therefore, the cell size is already reduced to 0.2 mm with the Behavior HARD option, which uses the rectangular shape of the area.
4. This area is the most crucial of the entire computational domain dealing with critical flow. Therefore, it has a double degree of adjustment. By default, all elements are set to 0.02 with the Behavior SOFT option for possible refinement in area 5 and triangle elements.
5. For mesh refinement in the nozzle area and nozzle output, the Sphere of Influence function was used. The sphere radius is 5 mm and the element size is 0.015 mm. This refinement also affects area 6.
6. Area 6 heads for the supersonic flow exiting from the nozzle. Therefore, by default, it has a cell size setting of 0.1 mm in Behavior SOFT mode and triangular elements.
7. The last area is adjacent to the area where the supersonic gas flow flowing from the nozzle is developed and subsequently braked. This area is already flow-calm but with a lower pressure than in area 1. Therefore, the cell size is set to 0.2 mm in Behavior HARD mode.
8. For the overall picture, it is necessary to add that areas with structured mesh have a Face Meshing setting with the Quadrilaterals method. Adaptive mesh refinement was used during the calculation. Sensitivity analysis was performed by multiple refinements of the mesh. Using manual adaptation using the field variable register by gradually increasing the value of the cells more than setting for the derivative option gradient for the static pressure variable led to a situation where the sensitivity analysis came out negative.

**Figure 11:**
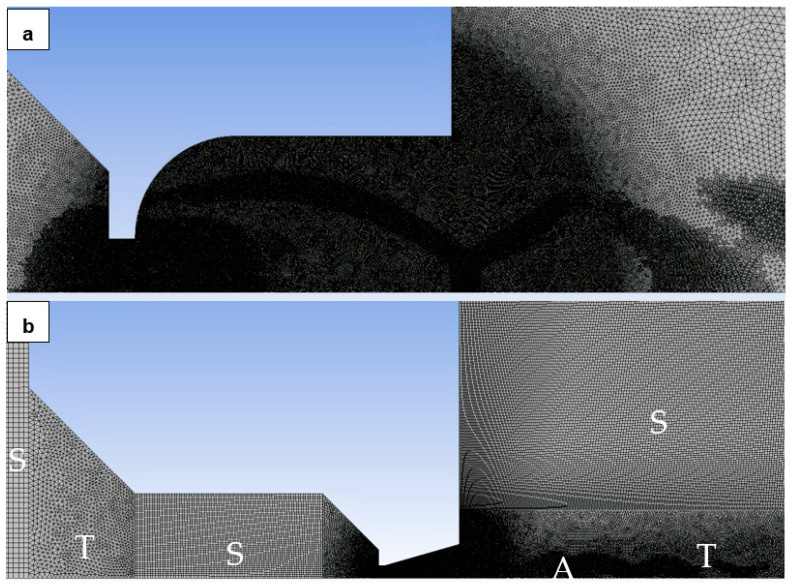
Zoomed area with the mesh refinement (**a**) and zoomed area with crucial points (**b**).

Figure 11a shows an increase in the area of the rectangular area (Figure 9—red box). The zoomed area shows the preplanned refinement. The refinement took place in places where oblique shock waves and perpendicular shock waves were formed [40].

Figure 11b shows a mesh image zoomed to the most crucial point of the computation in the aperture and nozzle area. The combination of structured mesh in Figure 11b marked S is evident in this figure. The mesh formed by triangles is shown in Figure 11b as a T. The areas adapted to the mesh during the calculation are also noticeable in Figure 11b, labeled A.

The range of mesh adaptation was determined based on maximum values in the cells’ derivative option, specifically focusing on gradient pressure with a maximum refinement level of 4. This approach effectively captured pressure gradients in the supersonic flow regions within the nozzle.

Prior analyses had already verified and fine-tuned the low-pressure boundary slip setting [41]. In Ansys Fluent, the Slip Flow mode was activated on the walls, respecting lower pressure conditions through Maxwell’s model:

(4) $$U_{w}-U_{g}=\left(\frac{2-\alpha_{v}}{\alpha_{v}}\right)K_{n}L_{c}\frac{\partial U}{\partial n}\approx \left(\frac{2-\alpha_{v}}{\alpha_{v}}\right)\frac{\lambda }{\delta }\left(U_{g}-U_{c}\right)$$

(5) $$V_{g}≣{\left(\vec{V}\vec{n}\right)}_{g}=V_{w}$$

where *U* and *V* are defined as components of velocity that are parallel and perpendicular to the wall. The indices *g, w*, and *c* indicate the gas velocities, the walls, and the center of the cell. *δ* is the distance from the center of the cell to the wall. *L_c_* is the characteristic length. *α_v_* represents the adjustment coefficient for the gas mixture, calculated as the mass-weighted average of each gas in the system. This option was selected to assess the solution using the mathematical Maxwell model under ESEM conditions, with an anticipated low-slip effect.

Furthermore, a grid independence study was conducted. Manual mesh adaptation was applied across the pressure range, utilizing a cell-in-range version for areas with minimal variable changes. The maximum refinement level was set at 2 for these regions, while, for areas preceding the aperture, within the nozzle, and in the gas expansion zone, a maximum refinement level of 4 was chosen.

The convergent criterion is set to continuity, x-velocity, y-velocity, and energy 1 × 10^−3^. At the same time, however, as will be discussed below, monitoring of the average value of static pressure, velocity, static temperature, and density in the entire computational volume has been set. Although the convergent criteria of the residuals were met, the calculation was carried out until the values of the monitors were equalized.

Additionally, parameter points were established at specific locations: within the aperture throat and at five points spaced 2 mm apart in the direction of gas flow above the aperture.

Similarly, the subsequent evaluations of quantities, as detailed later in the paper, exhibited consistency.

The grid independence analysis confirmed conformity, indicating that the mesh resolution was adequate for the type of analysis conducted.

## 4. Results and Discussion

The results presented in this paper will be the basis for the upcoming experiments. All of the research team is using a modern technique of a combination of experimental measurements with mathematical-physics analyses [42,43].

If the aperture is fitted with a nozzle, then the opening angle of the nozzle does not significantly affect the character of the gas discharge from the aperture, as demonstrated in the article [1]. However, if the lower part of the nozzle is opened in the shape of a cylinder, then the outlet from the aperture is significantly different than when the aperture is fitted with a nozzle, as can be seen below in the graphics of the Mach number (Figure 13), static pressure (Figure 17), and static temperature (Figure 18).

**Figure 12:**
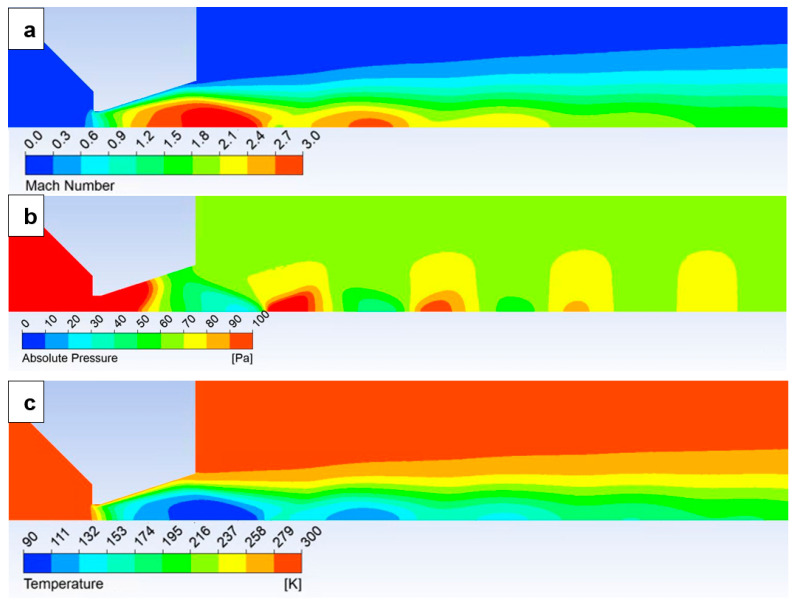
Mach number distribution (**a**), static pressure distribution (**b**), and static temperature distribution (**c**) of nozzle angle 18° variant.

The results show that all cylinder-based variants have almost identical results. The rounding of the bottom of the cylinder does not affect the static pressure. The shape of the lower corner of the cylindrical nozzle does not affect the character of the gas outlet from the nozzle and, thus, the distribution of Mach number (Figure 12a), static pressure (Figure 12b), and temperature (Figure 12c). Distribution of Mach number, static pressure, and temperature for other variants are shown in the figures in Appendix A. The gas in this area has a minimum velocity and does not affect the main supersonic flow.

As expected, the aperture-only variant and the original nozzle angle 18° variant are different from the cylinder variants.

**Figure 13:**
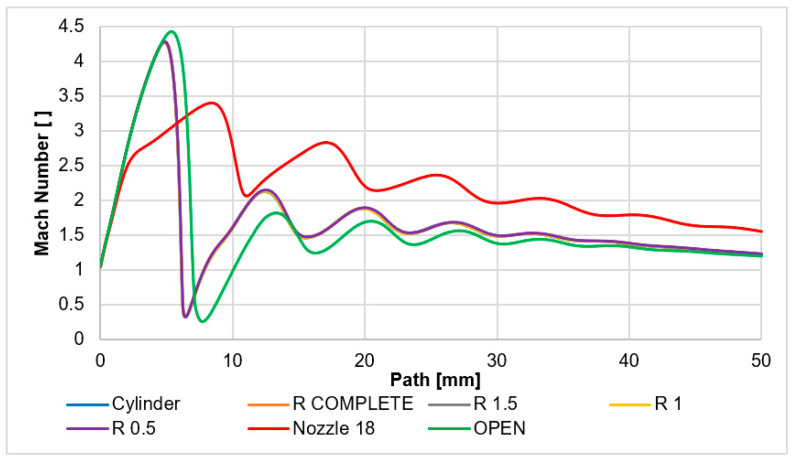
Mach number layout of each variant on path for each variant.

The Mach number will be analyzed first. This has a major impact on the course of static pressure and temperature. Figure 13 shows the Mach number course on the path.

In the nozzle angle of 18° variant, there is a noticeable overexpanded gas flow at the outlet from the nozzle. In the case of a nozzle with a larger opening than the calculated cross-section [1], the velocity course through the nozzle throat is uniform. However, it allows for overexpansion and, thus, gaining more velocity in the nozzle, but, in contrast, it allows for an earlier end of expansion behind the nozzle. It shows the drop in velocity in the nozzle itself. It is followed by a speed shift still in the area of the supersonic flow regime. This is also clearly visible in Figure 12a.

The course of the Mach number is completely different for the non-nozzle variants, i.e., the cylinder variant and the aperture-only variant (variants without any hint of a nozzle). There is an uncontrolled expansion reaching a velocity of over 4 Mach, followed by a sharp velocity drop to subsonic velocity. It is noticeable that the controlled expansion causes a slight increase in the Mach number as the gas is directed through the nozzle cone. However, the average velocity is higher than in the open variants on a longer path. It physically affects the other state quantities with which the Mach number is linked.

**Figure A1:**
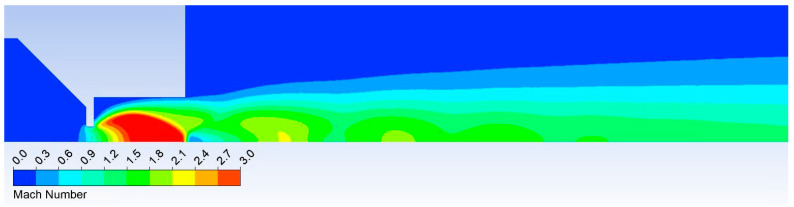
Mach number distribution of Nozzle Cylinder variant.

**Figure A2:**
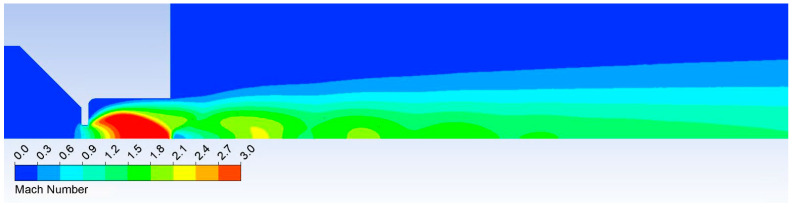
Mach number distribution of Nozzle Cylinder with R 0.5 mm variant.

**Figure A3:**
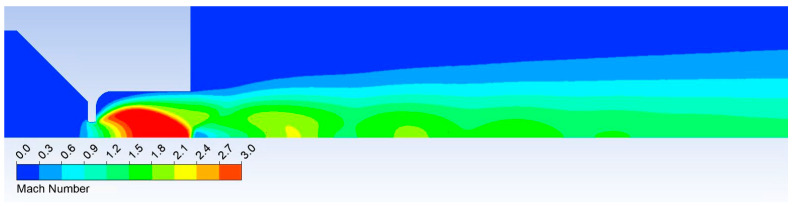
Mach number distribution of Nozzle Cylinder with R 1 mm variant.

**Figure A4:**
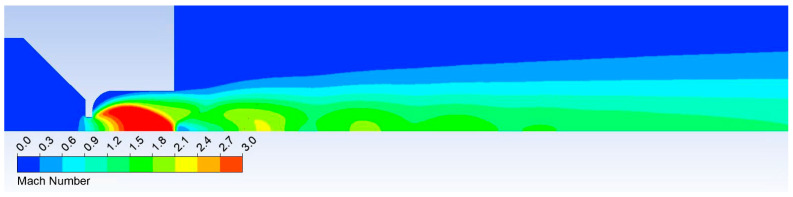
Mach number distribution of Nozzle Cylinder with R 1.5 mm variant.

**Figure A5:**
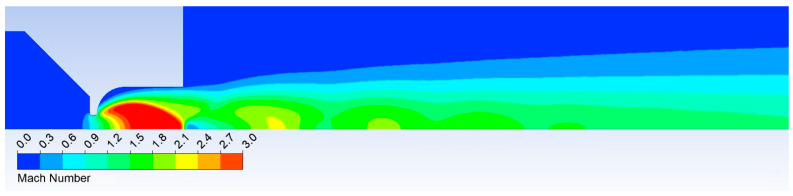
Mach number distribution of Nozzle Cylinder with R Complete variant.

**Figure A6:**
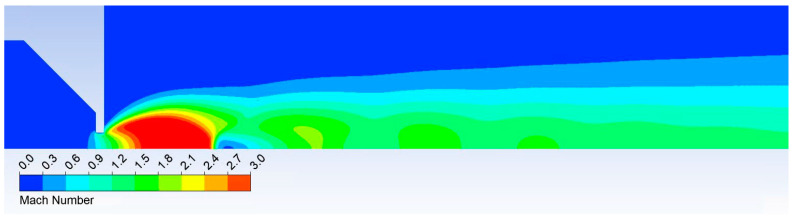
Mach number distribution of Nozzle-Only Aperture variant.

The Mach number distribution of the nozzle angle of 18° variant is shown in Figure 12a. The Mach number distribution of each variant can be seen in the figures in Appendix A (Figure A1, Figure A2, Figure A3, Figure A4, Figure A5 and Figure A6).

**Figure 14:**
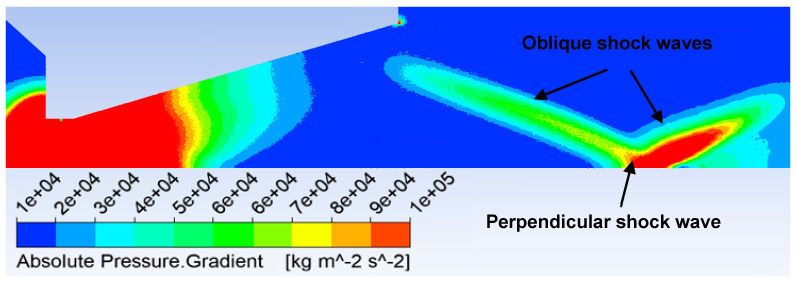
Pressure gradient distribution of nozzle angle 18° variant.

These differences in the course of the Mach number have a major impact on the formation of shock waves. Therefore, the knowledge of the pressure gradient distribution is suitable for describing the character of supersonic flow. At the same time, the pressure gradient affects the passage of the primary electron beam. Each region of increased pressure affects the scattering of the electron beam. Under the given conditions, a perpendicular shock wave and oblique shock waves are produced in each variant, as can be seen in Figure 14. Only in the cone variant is the perpendicular shock wave minimized at the expense of the oblique shock waves that come together at a given point.

**Figure A7:**
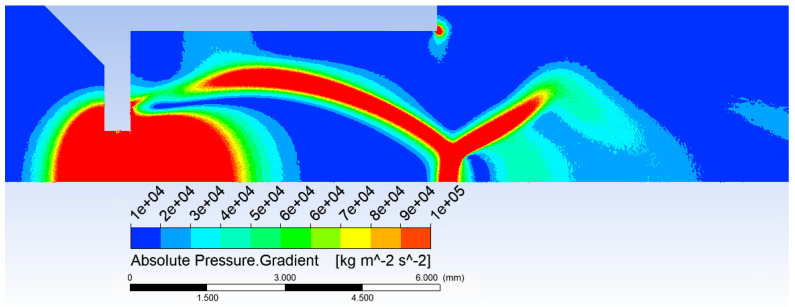
Pressure gradient distribution of Nozzle Cylinder variant.

**Figure A8:**
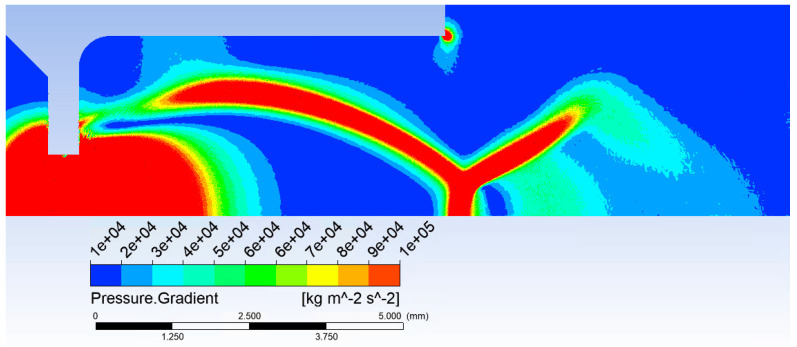
Pressure gradient distribution of Nozzle Cylinder with R 0.5 mm variant.

**Figure A9:**
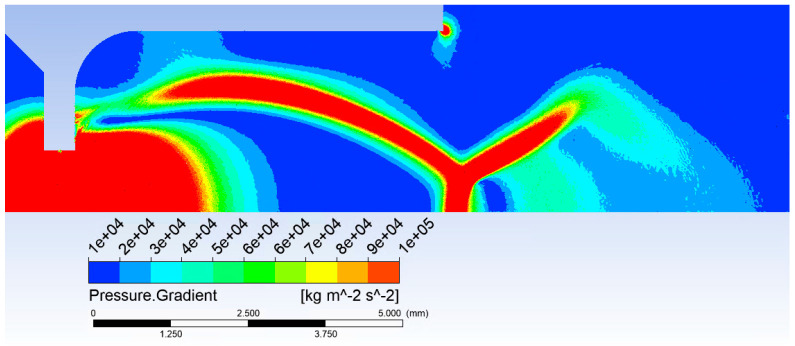
Pressure gradient distribution of Nozzle Cylinder with R 1 mm variant.

**Figure A10:**
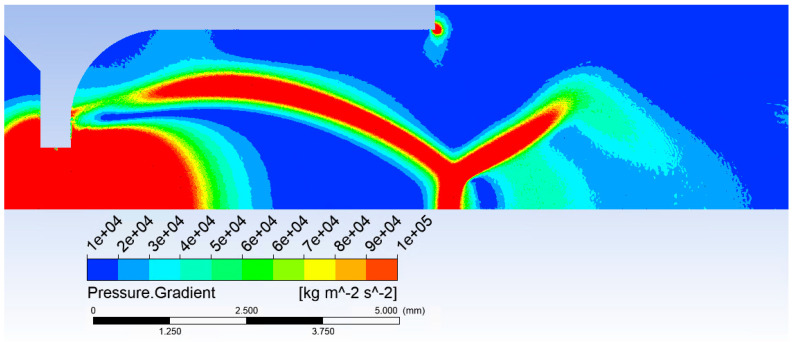
Pressure gradient distribution of Nozzle Cylinder with R 1.5 mm variant.

**Figure A11:**
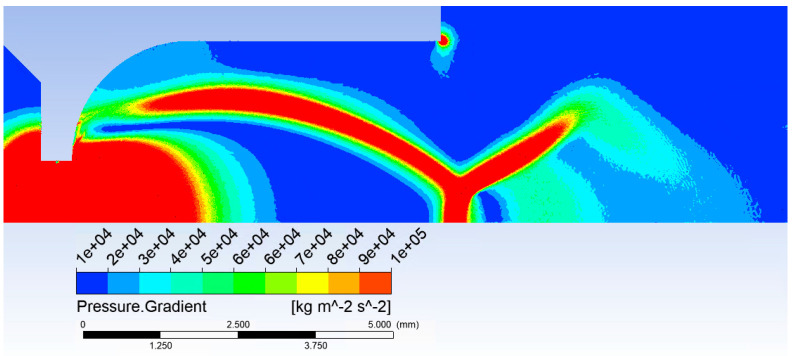
Pressure gradient distribution of Nozzle Cylinder with R Complete variant.

**Figure A12:**
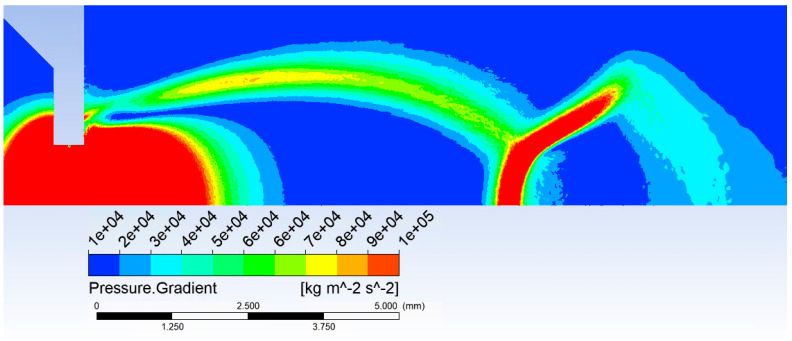
Pressure gradient distribution of Nozzle-Only Aperture variant.

The pressure gradient distribution of the nozzle angle of 18° variant is shown in Figure 14. The pressure gradient distribution of each variant can be seen in the figures in Appendix A (Figure A7, Figure A8, Figure A9, Figure A10, Figure A11 and Figure A12).

**Figure 15:**
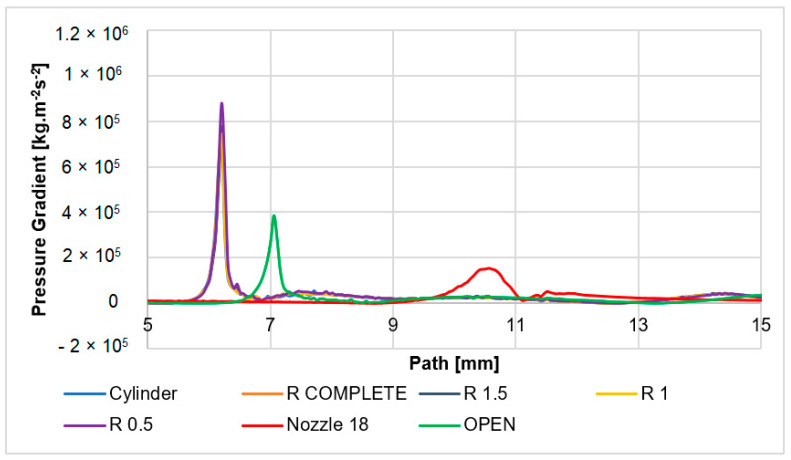
Pressure gradient layout of each variant on the path.

**Figure 16:**
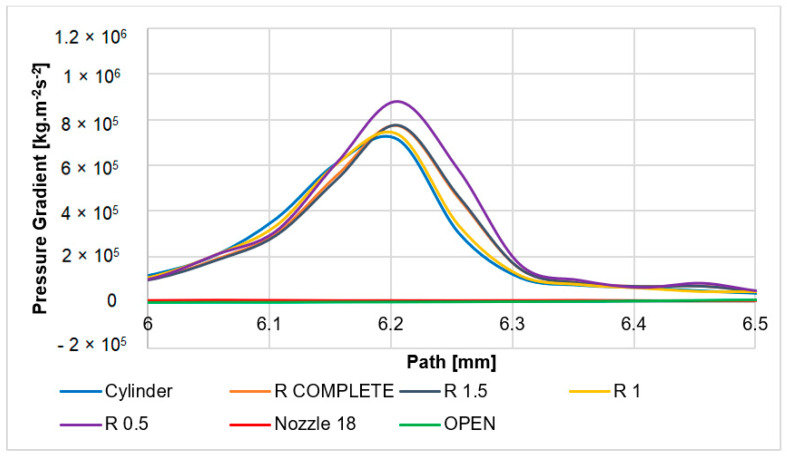
Pressure gradient layout of each variant in the limited scale on the path.

The overall distribution of the pressure gradient in the examined variants is shown and compared in Figure 15. The main gradient in all variants except the cone and open variants appears in a narrow space of perpendicular shock wave at one point but with a different intensity. For this reason, a small cut-out on the *x*-axis (path) in the limited scale of 6 to 6.5 mm was performed in Figure 16, which highlights the difference in gradient intensity in the individual variants.

With the nozzle angle of 18° variant, a very light perpendicular shock wave is evident compared to the other variants. Behind this, there is a slight increase in the gradient again, as was mentioned. Oblique shock waves are also weak, as they merge at the point of the perpendicular shock wave and thus weaken it. This weakened perpendicular shock wave emanates from all variants at the greatest distance from the aperture.

The open variant has a similarly different course compared to the other variants. Here, a more pronounced perpendicular shock wave is created but still of lower intensity than in the case of other variants. This is one of the reasons why oblique shock waves of lower intensity come out of a perpendicular shock wave.

Other variants have a very similar course of the perpendicular shock wave. Small differences in the intensity of the perpendicular shock wave are caused by the shaping of the lower part of the nozzle above the aperture. This affects the oblique shock waves, which follow the perpendicular shock wave in the upper part.

The physical reason why more significant perpendicular shock waves occur in the open variants is that it is a strongly overexpanded state of the nozzle. In selected cases of overexpanded open nozzles, the pressure distribution is created so that the back pressure from the chamber enters the nozzle and physically causes the formation of a perpendicular shock wave.

On the other hand, in the case of a nozzle with an angle of 18°, which is slightly overexpanded compared to the calculated state but significantly less than the open variants, it is still a controlled expansion. This nozzle creates oblique shock waves controlled at the outlet of the nozzle with the surrounding gas, but these are reflected inside the flow and reduce the expansion efficiency. For this reason, as mentioned, the flow velocity is lower than in the open variants. In addition, the reflected oblique shock waves connect in the axis of the flow and eliminate the formation of a perpendicular shock wave. In this case, when the nozzle is slightly overexpanded, a weak perpendicular shock wave is created. But, for the above reasons, it is significantly weaker compared to the open variants.

These facts have a significant impact on the static pressure distribution described below. These form the main obstacle to the passage of the primary electron beam. The static pressure curve corresponds to the Mach number course. Pressure will be captured using pressure sensors (multirange differential pressure sensors for gases and air DPS 300) in an experimental chamber [44,45]. This is controlled by the shape of the nozzle behind the aperture. The nozzle angle of 18° variant is significantly different from the other variants due to a smaller and more gradual increase in the Mach number (Figure 13). The shock wave occurs at a distance of 11 mm from the aperture. Due to the gradual increase in Mach number, there is not as much of a pressure drop behind the aperture as is evident in the other variants. A completely different process occurs with the open variant. This is the opposite of the controlled expansion in the nozzle angle of 18° variant. In this variant, there is a sharp increase in the Mach number and there is an even greater increase in the Mach number compared to other variants of the cylindrical type. Therefore, the subsequent drop occurs later. After that, the increase in Mach number back to supersonic velocity is lower than in the cylindrical variants. The reason for these facts is that the shape of the nozzle and the flow in the given nozzle physically significantly affect the state of pressure in the environment into which the flow from the nozzle enters. The open variants have a significantly different—higher—back pressure than the variant with a conical nozzle.

**Figure 17:**
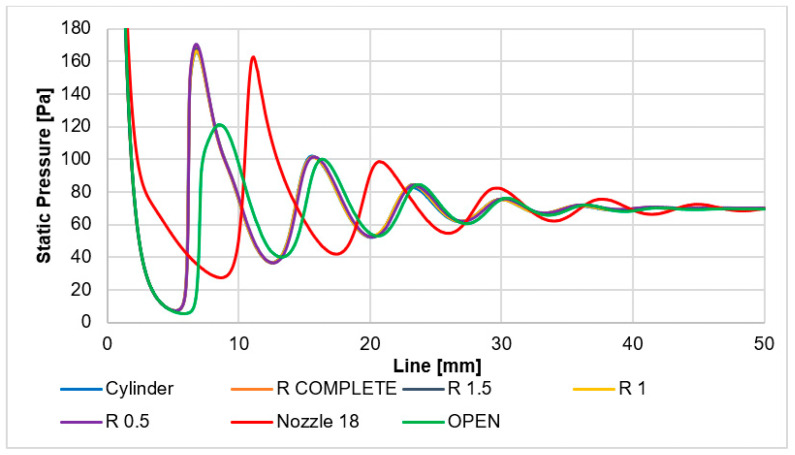
Static pressure layout of each variant on the path.

For this reason, the pressure gradient (Figure 15) is milder, as can also be seen in Figure 17. Here, the peak of the pressure curve in the 9–10 mm region is less sharp compared to the other variants. Therefore, the increase in pressure on the shock wave is also lower.

Other variants based on cylinder shapes are very similar. Due to a very sharp increase in the Mach number and then a sharp decrease, there are great pressure gradients. Therefore, the course of pressure also shows a sharp rise and drop in pressure. These facts will then be evaluated in connection with the expected electron scattering.

**Figure A13:**
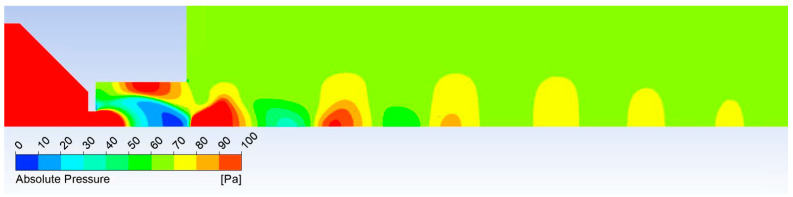
Static pressure distribution of Nozzle Cylinder variant.

**Figure A14:**
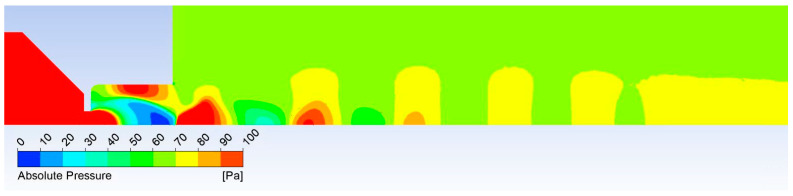
Static pressure distribution of Nozzle Cylinder with R 0.5 mm variant.

**Figure A15:**
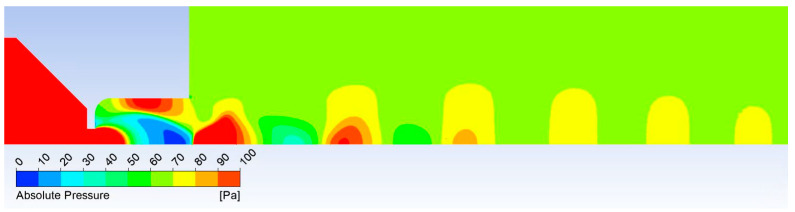
Static pressure distribution of Nozzle Cylinder with R 1 mm variant.

**Figure A16:**
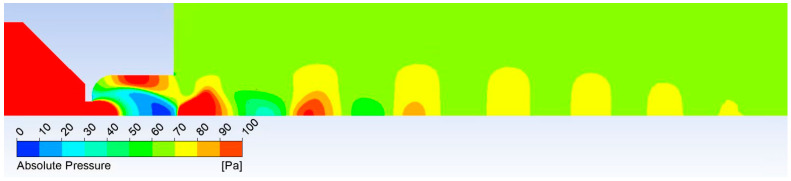
Static pressure distribution of Nozzle Cylinder with R 1.5 mm variant.

**Figure A17:**
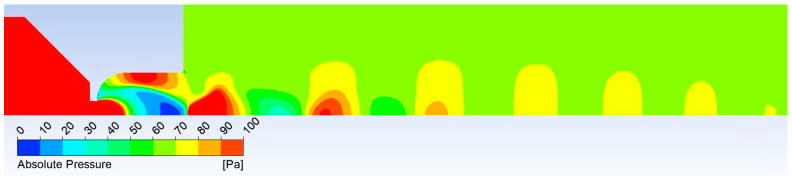
Static pressure distribution of Nozzle Cylinder with R Complete variant.

**Figure A18:**
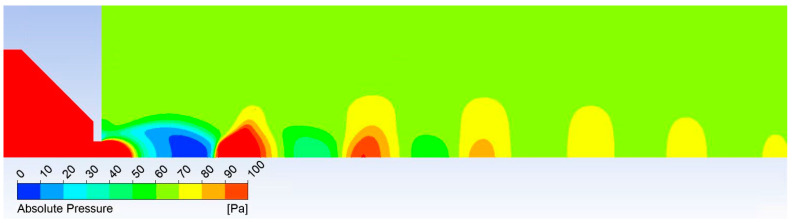
Static pressure distribution of Nozzle-Only Aperture variant.

The static pressure distribution of the nozzle angle of 18° variant is shown in Figure 12b. The static pressure distribution of each variant can be seen in the figures in Appendix A (Figure A13, Figure A14, Figure A15, Figure A16, Figure A17 and Figure A18).

**Figure 18:**
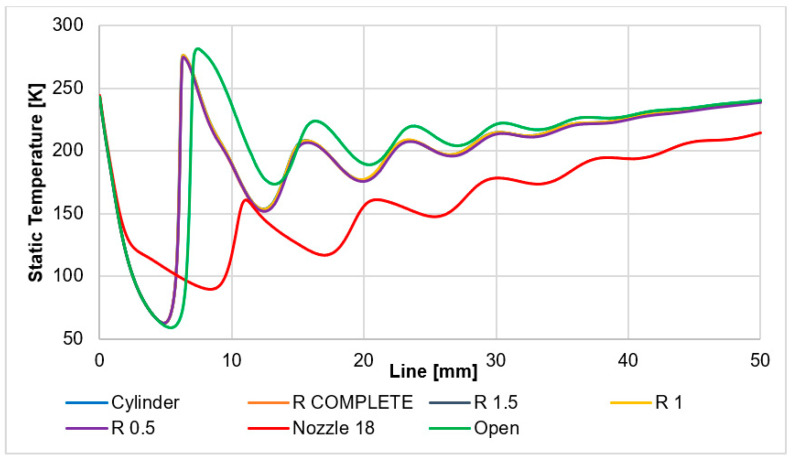
Static temperature layout of each variant on the path.

It is also advisable to analyze the course of static temperature on a given path when evaluating supersonic flow. The results are summarized in the graph in Figure 18.

**Figure A19:**
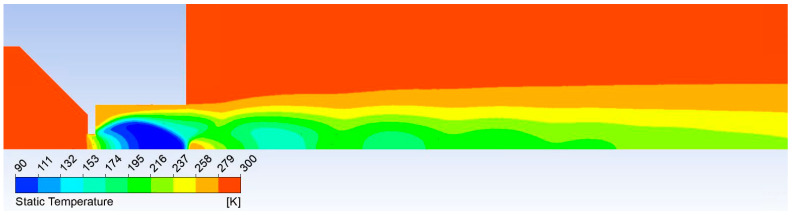
Static temperature distribution of Nozzle Cylinder variant.

**Figure A20:**
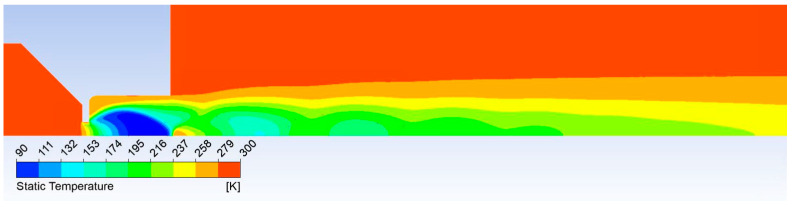
Static temperature distribution of Nozzle Cylinder with R 0.5 mm variant.

**Figure A21:**
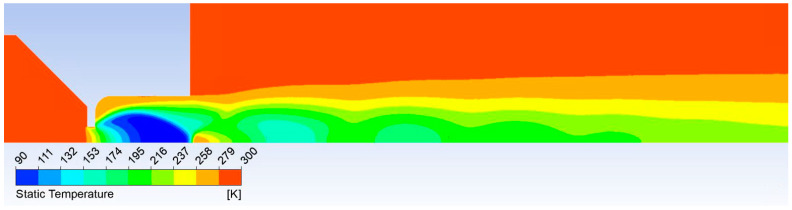
Static temperature distribution of Nozzle Cylinder with R 1 mm variant.

**Figure A22:**
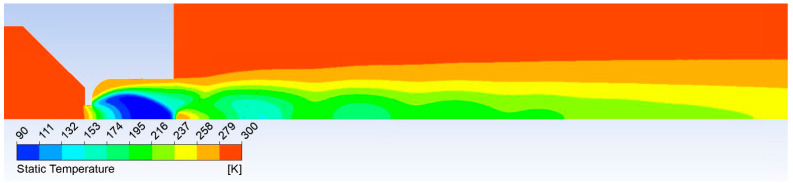
Static temperature distribution of Nozzle Cylinder with R 1.5 mm variant.

**Figure A23:**
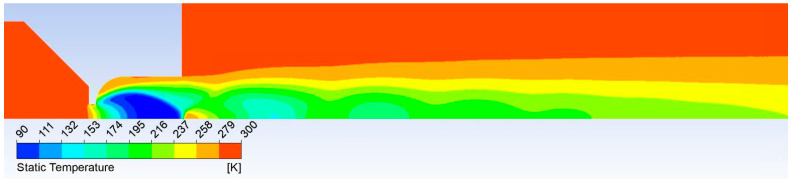
Static temperature distribution of Nozzle Cylinder with R Complete variant.

**Figure A24:**
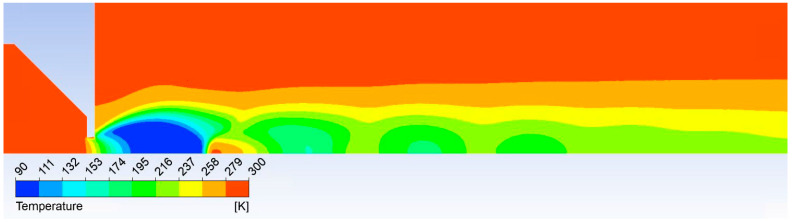
Static temperature distribution of Nozzle-Only Aperture variant.

The static temperature distribution of the nozzle angle of 18° variant is shown in Figure 12c. The static temperature distribution of each variant can be seen in the figures in Appendix A (Figure A19, Figure A20, Figure A21, Figure A22, Figure A23 and Figure A24).

The course of temperatures is analogous to the course of static pressure. This state quantity depends on static pressure as well as on Mach number. This knowledge will be used in the upcoming experimental measurements and will be scanned by the sensors (custom-made K-type thermocouple with a diameter of 3 mm).

### Evaluation of Electron Dispersion for Each Variant

Primary electrons are dispersed by the interaction of the beam and gas in an electron microscope. The operation of electron microscopes operating at higher pressure conditions in the specimen chamber (ESEM) is based on the fact that part of the beam electrons remain in their original path even after passing through the gaseous medium. This part of the electrons then interacts with the sample to create a signal, similar to a conventional electron microscope.

Electrons in the collisions can lose some of their energy and change the direction of their path. If the average number of collisions *M* in a gaseous medium is small, the resulting deviation of electrons from the original path of the beam in the plane of the preparation *r* is also small, and the length of the electron path can be laid equal to the thickness of the gas layer *d* through which the electron moves. The average number of collisions per electron can be determined from Equation (6) [46]:

(6) $$M=\frac{\sigma_{T}Pd}{kT}$$

where *σ_T_* is the total gas gripping cross-section, *P* is the static pressure, *d* is the thickness of the gas layer through which the electron passes, *k* is the Boltzmann constant, and *T* is the absolute temperature.

Then, for the value of *M*, the following applies:

- If *M* < 0.05, there is a minimum of beam dispersion of up to 5%;
- If *M* = 0.05–3, there is a partial dispersion in the range of 5–95%;
- If *M* > 3, there is a complete dispersion above 95%.

Gripping cross-section *σ_T_* is defined as the close area of a gas particle. If an electron is in this region as it passes by, a collision occurs. The gas gripping cross-section is, therefore, dependent not only on the type of gas but also on the accelerating voltage. This accelerating voltage is needed in the formation of the primary electron beam. Nitrogen gas and an accelerating voltage of 10 keV were chosen for this case. In this case, according to [47], the gripping cross-section *σ_T_* = 2 × 10^−21^ (m^2^).

**Figure 19:**
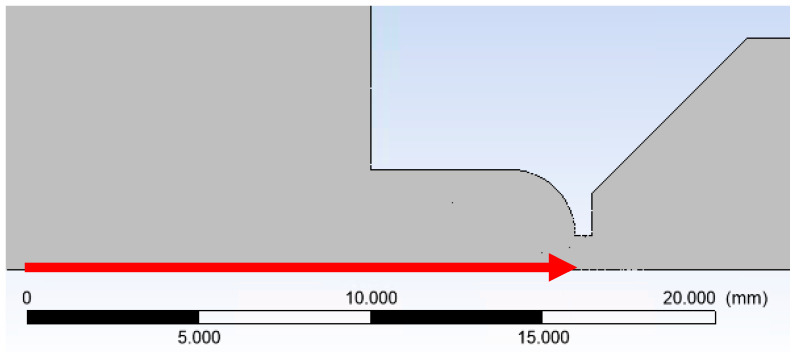
Direction of the primary electron beam path.

The path on which the primary electron beam passes through the nozzle in ESEM is shown in Figure 1 (axis). The primary electron beam runs against the flow direction, as indicated by the arrow in Figure 19.

**Figure 20:**
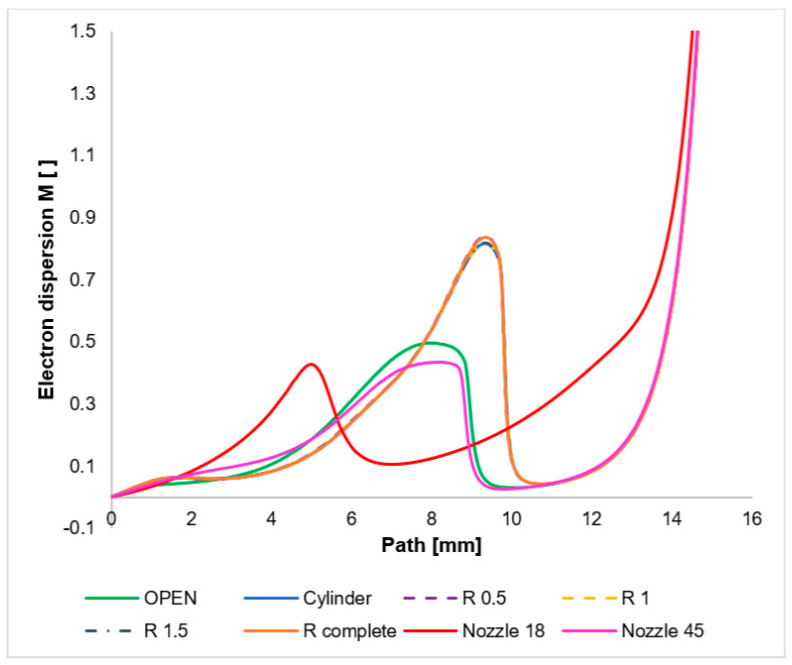
Course of electron dispersion value on the primary electron beam path.

The graph in Figure 20 shows the investigated scattering path with a length of 16 mm. This path is significantly longer than in the differential pumped chamber at ESEM. A usual path usually has an additional aperture at a distance of 5 mm. It separates the differentially pumped chamber from the tube, where there is a high vacuum.

The character of gas flow from nozzles of various shapes into free space is mapped in this paper. Furthermore, it is investigated how this changed character of flow affects the scattering of electrons. It is affected due to different shapes of shock waves. Based on this experience, an analysis will be performed for the actual shape of the differential pumped chamber in the next paper. Therefore, Figure 20 shows the distribution of the dispersion value *M* over a significantly longer path. For this reason, the values go up to high dispersion values. However, the purpose is to map the tendency of dispersion on shock waves.

The cylindrical character of the nozzles creates an overexpanded flow with a very significant perpendicular shock wave (Figure 14, Figure A7, Figure A8, Figure A9, Figure A10, Figure A11 and Figure A12). This forms an unsuitable barrier for passing electrons. It also affects the increased value of electron dispersion. In this case, the shaping of the lower part of the nozzle does not have a significant effect on the result of the shape of the shock waves (Figure 14, Figure A7, Figure A8, Figure A9, Figure A10, Figure A11 and Figure A12) and thus on the magnitude of electron dispersion (Figure 20). On the other hand, the 18° and 45° nozzles, which shape the supersonic flow cone behind the aperture, already have an effect. An angle of 18° will create an overexpansion and thus gain a lower velocity in the nozzle, as described in Figure 13. This has been described on a longer path than in cylindrical shapes; therefore, the shock wave at the end of the supersonic flow originates at a greater distance but is milder. For this reason, the electron dispersion is also half the value. There is a difference in the electron dispersion of the less open nozzles (18° and 45°) in the flow area behind the shock wave. With a smaller angle, there is a more significant re-increase in pressure and thus electron dispersion.

If, on the other hand, the completely open shape is used, a different flow character occurs than in the case of a cylindrical shape. As can be seen in Figure A12, this flow has less intense shock waves. Oblique shock waves have significantly less intensity and, similarly, perpendicular shock waves have less intensity, as can be seen in Figure A12. Therefore, even the curve describing the intensity of electron dispersion at a given location has approximately half the value of the electron dispersion intensity value of cylindrical shapes.

A curve for a 45° nozzle angle has been added to illustrate this. Its course is similar to that of the open variant. Thus, opening the nozzle above 45° has a similar effect as the open variant.

## 5. Conclusions

A combination of the experimental measurements using pressure sensors in conjunction with the theory of one-dimensional isentropic flow and mathematical-physics analyses was presented in this paper. This combination serves as a tool for the analysis and subsequent development of nozzles for environmental electron microscopy use, especially in the differential pumped chamber. An analysis of the impact of the shaping of the lower part of the nozzle on the character of the supersonic flow behind the aperture was performed. First of all, its effect on the shaping of oblique and perpendicular shock waves has a significant effect on the scattering of the electron beam. The purpose of this research is to ensure optimal pressure distribution in the axis of the primary electron beam, which passes through the environment of the differential pumped chamber, in such a way as to minimize the collision of the beam electrons with the gas molecules. It is, therefore, mainly a matter of ensuring the gas flow behind the aperture so that the pressure value is as low as possible. In this optimal distribution of gas pressure, the distribution of shock waves has a significant influence. Therefore, it was necessary to find such a shape of the nozzle that perpendicular shock waves would not appear in the primary beam path. In the first step, the character of the flow on the nozzle was first analyzed as a benchmark on the already fine-tuned mathematical-physics model for the selected pressure gradient *p_o_* = 2000 Pa and *p_v_* = 70 Pa for the nozzle shape, with a simplified shape of the Laval nozzle with an opening angle of 18°. Then, the expected Mach number course was evaluated using the theory of one-dimensional isentropic flow and, thus, the correctness of the mathematical-physics model was theoretically verified using this benchmark. From this benchmark shape onwards, subsequently, mathematical-physics analyses were performed using the Ansys Fluent system for selected nozzle shapes. The results showed that the cylindrical character of the nozzles creates an overexpanded flow with a very significant perpendicular shock wave. So, it creates an unsuitable barrier for passing electrons and affects the increased electron dispersion value. On the other hand, the conical nozzle shapes form the supersonic flow cone right behind the aperture. As a result of this nozzle shaping, the flow achieves an overall lower velocity in the nozzle. However, the drop in velocity to subsonic speed, in this case, occurs at twice the distance of cylindrical shapes. This has a very significant effect, as the shock wave at the end of the supersonic flow originates at a greater distance but is milder. Therefore, even electron dispersion has half the value when using this shape. The main difference in electron dispersion in the nozzle opening size occurs behind the shock wave. Here, a larger nozzle opening angle is more suitable for the nature of the flow for electron dispersion. A very special result for electron dispersion is the nozzle open shape. It combines the advantages of cylindrical shapes, where a sharp increase in dispersion values leads to a sharp decrease, and the advantages of a conical nozzle with a large opening angle, where there is not such a large peak of the dispersion value as in the case of a cylindrical shape. The character of free flow and limited flow in the nozzle and its influence on electron dispersion were also analyzed in this paper for ESEM research. These results of the flow nature concerning electron scattering are the starting point for further research. Further follow-up research will be directed for flow into a limited space with a free flow restriction, as is the case in a differentially pumped chamber.

## Data Availability

The data presented in this study are available on request from the corresponding author.
